# Feasibility testing of the Inspired Therapeutics NeoMate mechanical circulatory support system for neonates and infants

**DOI:** 10.1371/journal.pone.0266822

**Published:** 2022-05-11

**Authors:** Gretel Monreal, Steven C. Koenig, Mark S. Slaughter, Gino F. Morello, Steven R. Prina, Landon H. Tompkins, Jiapeng Huang, Barry N. Gellman, Kurt A. Dasse

**Affiliations:** 1 Department of Cardiovascular and Thoracic Surgery, University of Louisville, Louisville, Kentucky, United States of America; 2 Department of Bioengineering, University of Louisville, Louisville, Kentucky, United States of America; 3 Veritium Research LLC, Fort Lee, New Jersey, United States of America; 4 BLDC Designs LLC, Rocklin, California, United States of America; 5 Inspired Therapeutics LLC, Merritt Island, Florida, United States of America; 6 Department of Anesthesiology and Perioperative Medicine, University of Louisville, Louisville, Kentucky, United States of America; Policlinico S. Orsola-Malpighi, ITALY

## Abstract

Inspired Therapeutics (Merritt Island, FL) is developing a mechanical circulatory support (MCS) system designed as a single driver with interchangeable, extracorporeal, magnetically levitated pumps. The NeoMate system design features an integrated centrifugal rotary pump, motor, and controller that will be housed in a single compact unit. Conceptually, the primary innovation of this technology will be the combination of disposable, low-cost pumps for use with a single, multi-functional, universal controller to support multiple pediatric cardiopulmonary indications. In response to the paucity of clinically available pediatric devices, Inspired Therapeutics is specifically targeting the underserved neonate and infant heart failure (HF) patient population first. In this article, we present the development of the prototype Inspired Therapeutics NeoMate System for pediatric left ventricular assist device (LVAD) support, and feasibility testing in static mock flow loops (H-Q curves), dynamic mock flow loops (hemodynamics), and in an acute healthy ovine model (hemodynamics and clinical applicability). The resultant hydrodynamic and hemodynamic data demonstrated the ability of this prototype pediatric LVAD and universal controller to function over a range of rotary pump speeds (500–6000 RPM), to provide pump flow rates of up to 2.6 L/min, and to volume unload the left ventricle in acute animals. Key engineering challenges observed and proposed solutions for the next design iteration are also presented.

## Introduction

The development of mechanical circulatory support (MCS) devices for pediatric use has been greatly outpaced by adult devices, despite there being a critically unmet clinical need. Up to 14,000 children in the US are hospitalized with heart failure (HF)-related conditions each year with a mortality rate of 7–15% [1–3]. While 74% of children undergo heart transplantation within 90 days of listing [4]. the mortality rate while on the waiting list is 5–39% [3, 5, 6]; thus, both temporary and durable MCS devices play an important role in supporting this challenging patient population [6, 7]. Between the third and fourth Pedimacs reports, the use of MCS increased from 508 devices implanted [8] to 1031 [9, 10] through bridge to transplant (49% of pediatric assist device recipients) and bridge to candidacy (38%) strategies [9]. The majority of pediatric MCS recipients are those diagnosed with cardiomyopathy, followed by congenital heart disease (with 70% of hospital admissions in these patients occurring within the first year of life) and myocarditis [1, 9]. Actuarial survival of pediatric assist device recipients is 74% at 6 months, with age, severity of illness, and type of device used playing a complex role in patient outcomes [9].

The challenges in developing a suitable device for the pediatric population include the presence of congenital malformations, the etiology of cardiomyopathy, a wide variation in patient size and age, the potential for recovery, and the lack of data identifying the needs of the pediatric HF population. Limitations to current MCS technology include challenges with reducing the size of adult pumps to fit the small body habitus of the neonate and infant populations, potentially difficult cannula configuration that may need to work in the presence of congenital cardiac anomalies, challenging implant and management strategies (particularly if multiple devices (e.g. ventricular assist devices (VAD), extracorporeal membrane oxygenation (ECMO), cardiopulmonary bypass (CPB) are required for support), risk reduction (neurological complications, anticoagulation use), and cost effectiveness / cost reduction [11, 12].

To address these limitations, Inspired Therapeutics (Merritt Island, FL) is developing a novel MCS therapy, the NeoMate System, that will operate a family of interchangeable, single-use, extracorporeal, magnetically levitated (MagLev) pumps configured for multiple clinical indications. The NeoMate System is designed to combine all the driver components (integrated centrifugal pump, motor, controller) into a portable, wearable unit that supports the entire range of disposable products (pumps, cannulae) for cardiac and pulmonary indications. The NeoMate System is comprised of a single driver (controller with an embedded motor) with attachable components and is designed to support a family of MagLev pumps intended for short-term (up to 30 days) ventricular support (left and right), and cardiopulmonary support (ECMO, integrated respiratory assist, and cardiopulmonary bypass) for the pediatric population. In this article, we present development and testing of the prototype NeoMate System, intended to provide left ventricular assist device (LVAD) support for the neonate and infant HF patient population. The prototype NeoMate System was tested in static mock loop, dynamic mock loop, and acute ovine models to demonstrate feasibility.

## Materials and methods

### Development of the Inspired Therapeutics NeoMate System

**Fig 1:**
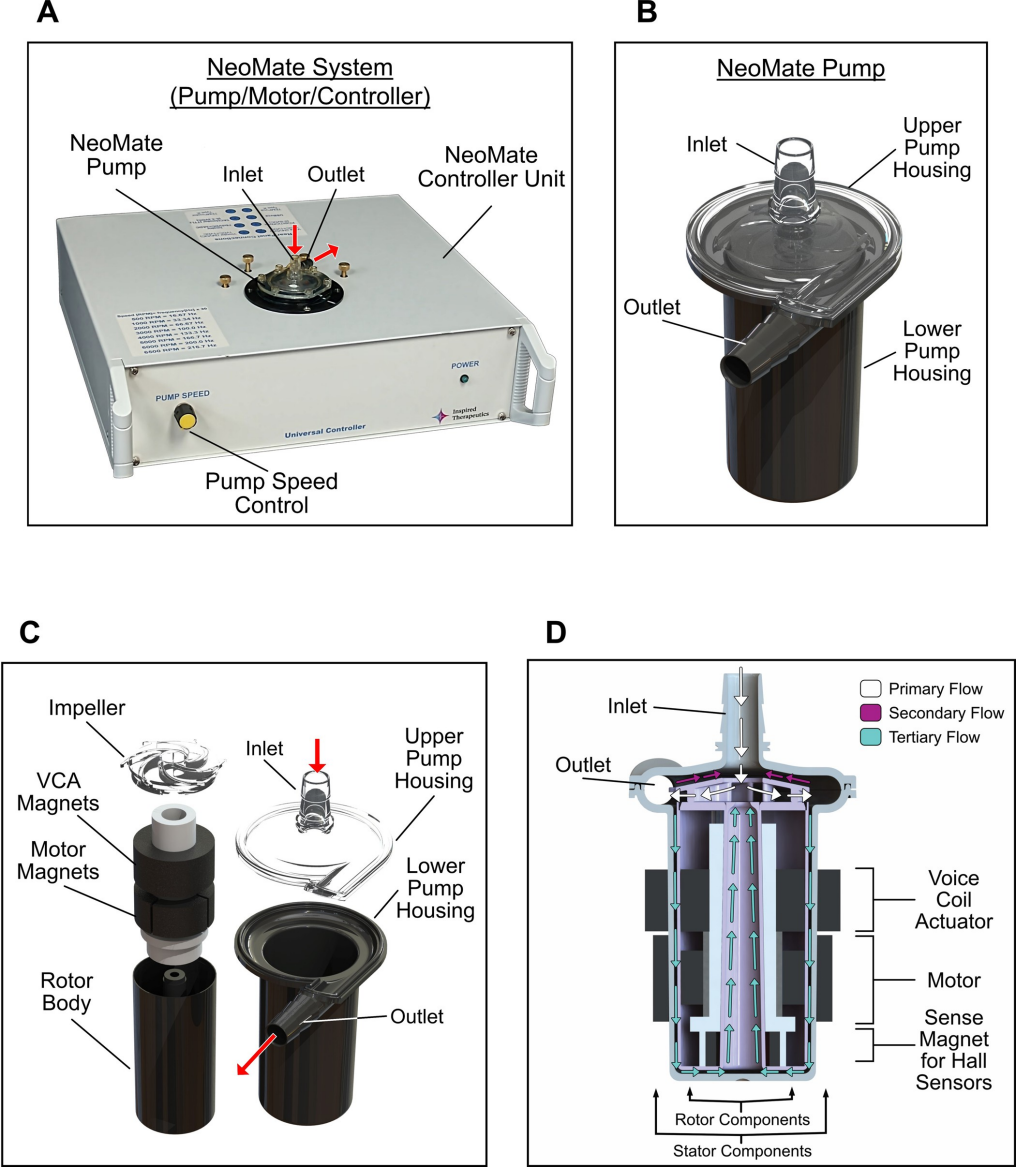
The prototype Inspired Therapeutics NeoMate System. (A) Prototype Inspired Therapeutics NeoMate System with integrated pump. (B) Pump housing, inflow, and outflow. (C) Exploded view of the pump and rotating group assembly. (D) Cross-section of the magnet, rotor, stator, voice coil actuator, and flow paths.

Three identical prototype NeoMate Systems (n = 3, herein referred to as Systems A, B, and C) each comprised of an integrated centrifugal pump, motor, and controller were fabricated, assembled, and tested for hydrodynamic performance and hemodynamic efficacy to demonstrate proof-of-concept and feasibility of preliminary design concept. The NeoMate System (**Fig 1A**) design criteria included demonstrating the ability to achieve flow rates of 0.5–3.5 L/min at pressures up to 150 mmHg with pump rotational speeds ranging from 500–6000 RPM. Initial design of the NeoMate pump impeller and preliminary CFD analysis based on these criteria have been previously reported [13]. Since this initial development, the impeller and pump designs have been iteratively improved, resulting in the first functioning shaft-less pump prototypes. A custom controller with integrated MagLev motor was developed to power the pump prototypes by combining an electric motor and active levitation system into a compact functional unit (**Fig 1A**).

#### Impeller, pump, and flow dynamics

Hydrodynamics of the NeoMate pump are achieved by an impeller mounted atop the MagLev rotor body to deliver centrifugal flow (**Fig 1B**). The impeller (27 mm diameter) contains six backward swept blades covered with a shroud designed to improve hydraulic efficiency (**Fig 1C**). The rotor body sits below the impeller and houses the rotating magnetic components of the MagLev system. A center opening (8 mm diameter) allows flow recirculation around the rotor body to counteract the lift forces generated by the thrust of the impeller during rotation to keep the impeller/rotor body assembly centered inside the pump housing and prevent axial contact. The impeller/rotor assembly is completely levitated and rotated inside the pump housing via the MagLev system (**Fig 1C**). The impeller was 3D printed using stereolithography (SLA) with a high-resolution ABS-like clear material (WaterShed XC), and the rotor body was fabricated with a high-density thermoplastic Ultem 1010 PEI (Polyetherimide, Performance Plastics). The pump housing surrounds the impeller/rotor and contains perpendicular barbed inlet and outlet ports (1/4” diameter) along the pump volute for cannula attachment. The stationary components of the MagLev system were mounted to the exterior of the pump housing. The pump housing (14.5 mL priming volume) was split into upper and lower sections to facilitate prototype assembly. The upper pump housing was SLA printed using an ABS-like clear material (WaterShed XC) to facilitate flow visualization, and the lower pump housing was fabricated with Ultem 1010 PEI (Polyetherimide, Performance Plastics). There are three distinct flow paths within the pump (**Fig 1D**): (1) primary path with flow entering the inlet of the pump housing, then into the impeller eye, through the impeller blades and into the volute, and exiting at the pump outlet; (2) secondary path with flow down the outside of the rotor body and up through the center opening of the rotor body and impeller; and (3) tertiary path at the top of the impeller shroud and the upper pump housing where flow reverses and travels back toward the jet flow at the pump inlet. The secondary and tertiary flow paths are designed to wash the pump surfaces to help reduce stagnate flow and prevent thrombus formation.

#### Universal controller

The controller enclosure (**Fig 1A**) houses the prototype integrated pump, MagLev motor, and all control electronics of the NeoMate System. The electric motor and levitation system were designed to be capable of suspending and rotating the impeller/rotor assembly within the centrifugal blood pump without contact during clinical use. MagLev is accomplished using a voice coil actuator (VCA) with active longitudinal axial positioning and radial stability from the surrounding fluid. Four primary subsystems were developed: (1) 24 VDC isolated power supply with universal alternating current (AC) input, (2) sinusoidal motor controller for rotational control of the impeller/rotor, (3) linear VCA closed-loop position controller for longitudinal position control of the impeller/rotor during levitation, and (4) Hall sensor array for longitudinal position measurement of the impeller/rotor. The prototype systems are also designed with rear panel analog and digital outputs and input for connection to external data acquisition systems, test equipment, and control computers. The rotating components of the motor and levitation system are contained inside the rotor body and the stationary components are contained on the exterior of the pump housing, inside the controller enclosure. The impeller/rotor’s motor is a slot-less brushless DC design and operates over the rotational speed range of 500 RPM to 6000 RPM. After calculating and empirically confirming the torque requirements of the pump to achieve the required flows and pressures [14], a full-custom motor and linear VCA were designed. The system’s power supply is a commercial-off-the-shelf (COTS) design with a universal AC input and 24 VDC output, which provided the current required for longitudinal position control during lift-off and for operating the motor over the required speed range. A sensorless sinusoidal motor controller is used for start-up, open-loop commutation, closed-loop commutation, and speed control. The motor controller and driver are designed to source continuous currents up to 2.0 ADC and short duration peak currents up to 3.0 ADC. Pump speed is controlled via a manual multi-turn speed control knob mounted on the front panel of the controller and is used to limit the slew rate of the impeller/rotor speed. The active longitudinal position control system uses a solenoidal VCA, a permanent sense magnet installed at the base of the rotor assembly, and a Hall effect sensor array located in the base of the pump module receptacle within the controller. The Hall sensor array is aligned to sense the longitudinally oriented magnetic field of the rotor permanent sense magnet and used to monitor instantaneous longitudinal position of the pump rotor. The longitudinal position control system uses the Hall sensor output, which is directly proportional to the instantaneous proximity to the permanent magnet in the rotor to modulate the current in the VCA. This approach functions to maintain the longitudinal position of the impeller/rotor at 0.040” tolerance; however, due to the compact packaging of the magnetic components within the rotor, the VCA was found to contribute peak magnetic fields of ±11mT on top of the sense magnet field yielding poor signal-to-noise ratio. To mitigate this effect, the voltage as a function of VCA instantaneous current was subtracted from the voltage generated by Hall position array to improve the signal-to-noise ratio. A proportional integral derivative (PID) control loop was designed to levitate motor and maintain stable position over the entire operating range.

### Prototype testing

To demonstrate feasibility of the prototype NeoMate System, two Systems (A and B) were tested in static mock flow loops (H-Q curves), dynamic mock flow loops (hemodynamics), and in an acute healthy ovine model (hemodynamics, surgical feasibility, clinical applicability) and one System (C) was bench tested for long-term reliability. Each of the three Systems retained their same components (impeller, shroud, housing, etc) throughout all the testing.

#### Static mock flow loop model testing

Static mock loop experiments were performed to quantify *in vitro* pump hydraulic performance (head pressure (H) to flow (Q), H-Q curves) of the NeoMate System. Two prototypes (System A and System B) were tested using three different pairs of inflow and outflow cannulae. Six static mock loop experiments were performed in total. Both Systems A and B were each tested in the static mock loop using three different blood-analog volumes and pairs of pediatric cannulae (Medtronic, Minneapolis, MN). The mock loop volumes (with corresponding cannulae pair) were chosen to simulate the blood volume size equivalence to a neonate, infant, and toddler. The volumes (reservoir plus loop prime volume) and cannula pairs tested, from smallest to largest size, were as follows: 1) 300mL volume, inflow cannula 68112, 15”, 12Fr; outflow cannula 77110 9”, 10Fr. 2) 500mL volume, inflow cannula 68116, 15”, 16Fr; outflow cannula 77112, 9”, 12Fr. 3) 2400mL volume, inflow cannula 68124, 15”, 24Fr; outflow cannula 77116, 9”, 16Fr. Pressure gradient and flow data were collected over a range of pump speeds by varying outflow resistance.

**Fig 2:**
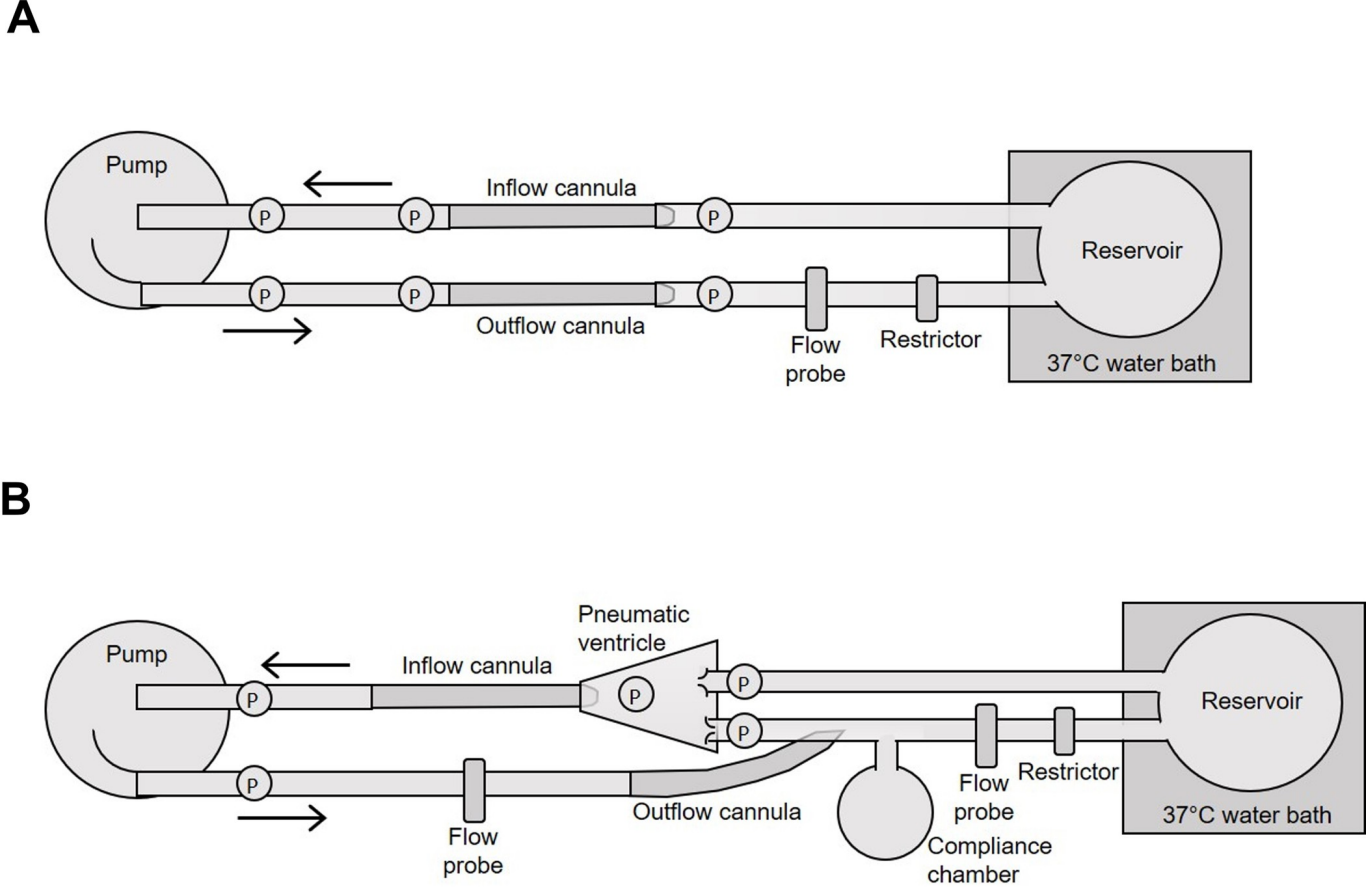
Mock flow loop models. Illustration of static (A) and dynamic (B) mock flow loop models for testing hydrodynamic and hemodynamic performance of the prototype Inspired Therapeutics NeoMate System. P, pressure sensor.

The static mock loop was designed as shown in **Fig 2A**. The NeoMate System was connected to the mock loop circuit using ¼” tubing (ND-100-65 Tygon PVC, ¼”, 8349T42, McMaster-Carr, Elmhurst, IL). A glycerol-water solution of 3.8 cP at 37°C was prepared to mimic the viscosity of blood and validated using a Cannon-Fenske viscometer (7988–37, size 75, ACE Glass, Vineland, NJ). A Hoffman clamp placed on the mock loop tubing was used to adjust the outflow resistance. The mock loop reservoir was placed inside a water bath to maintain the circuit at 37°C. Six fluid-filled transducers (MLT0380/D, ADInstruments, Colorado Springs, CO) were used to acquire pressure data at the inlet and outlet of the pump head, and then both proximal and distal to the inflow and outflow cannulae. A 7mm flow probe (Transonic Systems, Ithaca, NY) was placed on the pump outflow tubing. Hemodynamic data from the pressure transducers and flow module (TS410, Transonic Systems) were collected using a PowerLab 16/35 acquisition system with bridge amps (ADInstruments). Pressure transducers were calibrated using an external manometer (Meriam, Cleveland, OH). Controller output parameters, motor voltage (1 VDC = 1 VDC), motor current (1 VDC = 3 ADC), and motor temperature (4mV/°C), were measured and power was calculated. Data were recorded using LabChart v.8.1.16 (ADInstruments) at a sampling rate of 1000/s.

Once the static mock loop was primed with the glycerol-water solution and de-aired, the pump was set to 500 RPM and no resistance. Flow was then decreased in 0.2 L/min increments by increasing outflow resistance (Hoffman clamp) and data were collected at each increment. Pump speed was then increased by 500 RPM increments up to 5000 RPM, and the stepwise data were reacquired at each RPM increment. Data were collected from each of the 6 pressure transducers, the flow probe, and from the controller intrinsic parameters. Delta pressure was calculated across the pump (ΔP pump as measured via the pressure transducers at the pump inlet and outlet, **Fig 2A**) and across the circuit (pump plus cannulae, ΔP circuit as measured via the pressure transducers post-outflow cannula and pre-inflow cannula, **Fig 2A**). The above experimental design was repeated for each controller and each cannula pair. Mean values for each parameter at each test condition were calculated and resulting H-Q curves were plotted using Prism v.9.1.2 (226) (GraphPad, San Diego, CA).

#### Dynamic mock flow loop model testing

Dynamic mock loop experiments were performed using a pneumatic mock ventricle to quantify the *in vitro* hemodynamic performance of Systems A and B. Two single left-sided dynamic mock loop experiments were performed. Both Systems A and B were each tested in the dynamic mock loop using a single pair of pediatric cannulae. The cannula pair tested was inflow cannula 68124, 15”, 24Fr; outflow cannula 77116, 9”, 16Fr (both Medtronic, the largest pair tested in the static loop) with a blood-analog volume of 3600mL.

The dynamic mock loop was designed as shown in **Fig 2B**. The pump and controller were connected to the mock loop circuit using ¼” tubing (ND-100-65 Tygon PVC, ¼”, 8349T42, McMaster-Carr). The outflow cannula was connected to the apex of a silicone ventricle powered by a pneumatic driver (Ventricular Assist Device Pneumatic Drive System, Thoratec, Pleasanton, CA (now Abbott)). Bioprosthetic valves (Mosaic 305 Cinch II, 23mm, Medtronic) were placed in the aortic and mitral positions. The pump outflow cannula fed into the aortic position via a large Tuohy (22828, Qosina, Ronkonkoma, NY). A glycerol-water solution of 3.8 cP at 37°C was prepared to mimic the viscosity of blood and validated using a Cannon-Fenske viscometer (7988–37, size 75, ACE Glass). A Hoffman clamp placed on the mock loop tubing was used to adjust resistance as needed. A 1L compliance chamber was placed proximal to the reservoir, and the reservoir was placed inside a water bath to maintain the circuit at 37°C. Five fluid-filled transducers (MLT0380/D, ADInstruments) were used to acquire pressure data at the inlet and outlet of the pump head, the left ventricle, the aorta, and the venous position of the dynamic mock loop. A 7mm flow probe (Transonic Systems) was placed on the pump outflow tubing. Hemodynamic data from the pressure transducers and flow module (TS410, Transonic Systems) were collected using a PowerLab 16/35 acquisition system with bridge amps (ADInstruments). Pressure transducers were calibrated using an external manometer (Meriam). Controller output parameters, motor voltage (1 VDC = 1 VDC), motor current (1 VDC = 3 ADC), and motor temperature (4mV/°C), were measured and power was calculated. Data were recorded using LabChart v.8.1.16 (ADInstruments) at a sampling rate of 1000/s.

Once the dynamic mock loop was primed with the glycerol-water solution and de-aired, the mock loop was tuned to a heart rate of 150 bpm, mean aortic pressure of 50 ± 5 mmHg, mean aortic flow of 2.0 ± 0.2 L/min, and venous pressure of 15 ± 5 mmHg. Hemodynamic and pump/controller data were acquired with the pump off and clamped, the pump off and unclamped, and then with the pump on at speeds of 500 to 5000 RPM, increased in 500 RPM increments. Data were analyzed using the LabChart Blood Pressure Module (ADInstruments) and plotted using Prism (GraphPad).

#### Bench testing

Prototype System C was used in an impromptu test for long-term reliability of the MagLev motor and controller electronics using a static flow loop (different static loop design than described above in part B) with pump inflow and outflow connected to ¾” Tygon tubing via ¼” to ¾” step-up connectors and connected to a static reservoir (20 L volume). The system continues to operate at 500 RPM to provide a flow rate of 0.25 L/min in a temperature-controlled environment (23°C ± 2°C). Low speed, pressure, and flow rate were intentionally used as components of the pump are made from 3D printed material for which the long-term performance and durability are not known.

#### Acute ovine model testing

All animals used were housed in a facility in accordance with the Association for Assessment and Accreditation of Laboratory Animal Care (AAALAC). The testing protocol (#18393) was approved by the Institutional Animal Care and Use Committee (IACUC) at the University of Louisville. This investigation and all efforts to reduce suffering via sedation, anesthesia, analgesia, and euthanasia are described in detail below conform to the National Research Council. 2011. Guide for the Care and Use of Laboratory Animals: Eighth Edition. Washington, DC: The National Academies Press.

Two acute (non-survival) sheep studies were performed to assess pump performance of Systems A and B to demonstrate early prototype system feasibility *in vivo* (hemodynamics, surgical feasibility, clinical applicability). Sheep were selected as the animal model, as future chronic survival studies will require externalized cannulae and the use of a jacket to house the Inspired System, and thus an animal model compatible with having externalized components and being tethered and halter-led (unlike pigs) was selected. Two sheep (40.5kg and 43kg Dorset ewes, Noble Life Sciences Inc, Woodbine MD) were sedated with xylazine (0.04 mg/kg IM) and a catheter was placed in an ear vein. Propofol (4 mg/kg IV) was administered, the animals were endotracheally intubated, and an orogastric tube was placed for rumen decompression. Mechanical ventilation was initiated, and tidal volume and respiratory rate were adjusted to maintain a PaCO_2_ of 35–45 mmHg under general isoflurane anesthesia (1–3%). The animals were positioned in right lateral recumbency. A transesophageal echocardiography (TEE) probe (X7-2t transducer with Philips iE33, Philips Healthcare, Andover, MA) was placed for intraoperative monitoring and data acquisition/analysis. 0.9% saline was infused for maintenance as needed (5–10 mL/kg/hr IV). Three-lead electrocardiography, body temperature, pulse oximetry, and end-tidal CO_2_ were continuously monitored. Rocuronium (1.2 mg/kg IV) was administered just prior to incision. A small incision was created in the left neck for the placement of 7Fr introducers in the left carotid artery and left jugular vein for pressure monitoring, blood draws, and/or drug administration if needed. A thoracotomy was performed at the 5^th^ intercostal space and the 5^th^ rib was removed. The pericardium was opened anterior to the phrenic nerve and pericardial stay sutures were placed. A 16mm flow probe (Transonic Systems) was placed around the pulmonary artery (PA) trunk. Pursestring sutures secured fluid-filled pressure lines into the left atrium, right atrium, aorta, and left ventricle for hemodynamic data collection.

Baseline data (pre-LVAD) were acquired with ventilation temporarily suspended. Data included blood chemistry and oximetry (i-STAT, Abbott Point of Care, Chicago, IL), activated clotting time (ACT; ACT II, Medtronic, Minneapolis, MN), plasma free hemoglobin (HemoCue, Ängelholm, Sweden; modified azidemethemoglobin reaction), TEE, hemodynamics from the pressure transducers and PA flow probe, and intrinsic pump parameters. Comprehensive TEE was performed, left and right ventricular systolic and diastolic function was evaluated, all valves were examined for regurgitation or stenosis, and Simpson’s disk method was used to calculate left ventricular volume. Hemodynamic data from the fluid-filled pressure transducers (MLT0380/D, ADInstruments) and flow modules (TS420, Transonic Systems) were collected using a PowerLab 16/35 acquisition system (ADInstruments) with bridge amps (ADInstruments FE224). Pressure transducers were calibrated using an external manometer (Meriam). Controller output parameters, motor voltage (1 VDC = 1 VDC), current (1 VDC = 3 ADC), and temperature (4mV/°C) were measured, and power was calculated. Data were recorded using LabChart v.8.1.16 (ADInstruments) at a sampling rate of 400/s.

The animals were heparinized to achieve an ACT >400s. The pump and tubing were primed with saline and clamped. The pump outflow cannula (77116, 9” length, 16Fr, Medtronic) was placed in the proximal descending aorta. Pledgeted pursestring sutures were placed in the left ventricular apex and the pump inflow cannula (68124, 15” length, 24Fr, Medtronic) was placed into the apex through a small cruciate incision. TEE was used to verify that the inflow cannula was pointed toward the aortic valve and was parallel to the interventricular septum. A 2–3” section of Hemashield graft (Getinge, Göteborg, Sweden) bridged each cannula to the pump tubing (ND-100-65 Tygon PVC, ¼”, 8349T42, McMaster-Carr) to provide a site for atraumatic clamping. A 7mm flow probe (Transonic Systems) was placed on the pump outflow tubing. Fluid-filled pressure transducers were connected to the pump inflow and outflow tubing. The pump and circuit were primed with saline and de-aired, the cannulae were back bled, and the lines were temporarily re-clamped in preparation for the first experimental dataset.

System A was tested on the first sheep and System B was tested on the second sheep. All data collected at baseline (bloodwork, TEE, hemodynamics, pump/controller data) were repeated for each pump setting to be tested. Each dataset consisted of a hemodynamic recording period of ~10–15 seconds with the ventilator suspended and an acclimatization period of 5–10 minutes between each RPM increase prior to each recording. The datasets recorded included: 1) Pump off and clamped; 2) Pump off and unclamped; 3) 3000 RPM; 4) 4000 RPM; 5) 5000 RPM; 6) 6000 RPM. Following completion of the study protocol, the animals were euthanized under general anesthesia with Beauthanasia-D (0.22 mg/kg IV). Necropsies were performed for examination of the pump, cannulae, heart, great vessels, and end-organs.

Data were analyzed using the LabChart Blood Pressure Module (ADInstruments) and plotted using Prism (GraphPad). Hemodynamic data from the animal tests are presented as the mean of 10–20 cardiac cycles worth of data at each test condition.

## Results and discussion

### Static mock loop model

**Fig 3:**
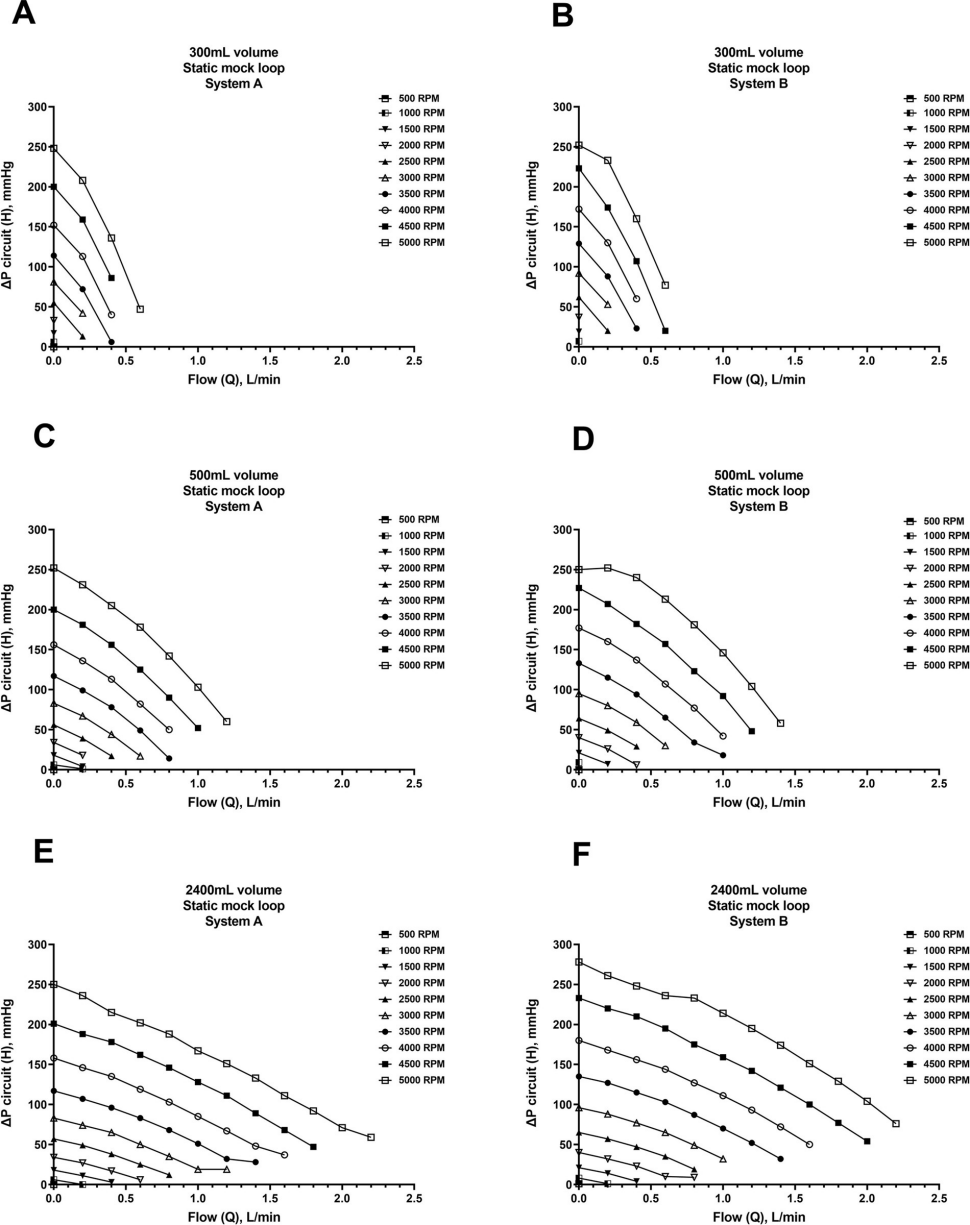
Static mock flow loop data–ΔP circuit (pump and cannulae). Relationship of the circuit head pressure (H) to flow (Q) (ΔP pump plus cannulae) for the Inspired Therapeutics NeoMate System in a static mock flow loop model over a range of pump speeds (RPM) and outflow resistances. H-Q curves for Systems A and B and three pairs of inflow and outflow cannula were generated (A-F).

**Fig 4:**
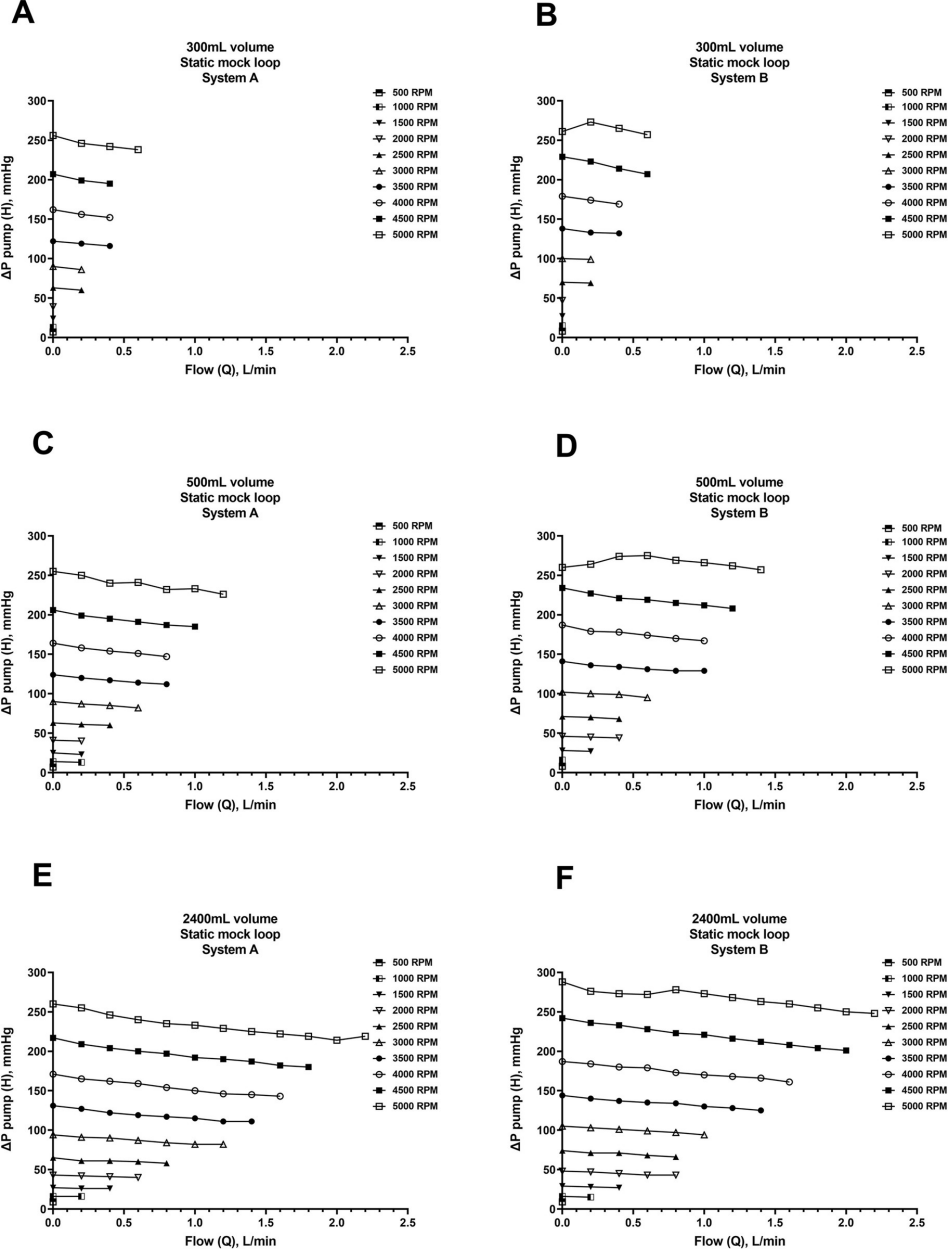
Static mock flow loop data—ΔP pump (inlet and outlet). Relationship of pump head pressure (H) to flow (Q) (ΔP pump inlet and outlet pressures) for the Inspired Therapeutics NeoMate System in a static mock flow loop model over a range of pump speeds (RPM) and outflow resistances. H-Q curves for Systems A and B and three pairs of inflow and outflow cannula were generated (A-F).

Experiments were performed to quantify *in vitro* pump hydraulic performance (H-Q curves) of Systems A and B using three different pairs of inflow and outflow cannulae. The results from the six static mock loop experiments demonstrated both Systems A and B performed comparably to each other. The static mock loop data graphed in **Figs 3 and 4** are described using the mock loop volume rather than the cannulae pair, as a future goal of the NeoMate System is to accommodate a wide range of commercially available cannulae based on patient size, anatomy, and clinical need. In the 300mL volume mock loop (smallest cannulae pair: inflow cannula 68112, 15”, 12Fr; outflow cannula 77110, 9”, 10Fr), both Systems A and B delivered up to 0.6 L/min flow against a ΔP circuit of 50-75mmHg at 5000 RPM (**Fig 3A and 3B**) and up to 0.5 L/min flow against a ΔP pump of 250mmHg at 5000 RPM (**Fig 4A and 4B**). In the 500mL volume mock loop (intermediate-sized cannulae pair: inflow cannula 68116, 15”, 16Fr; outflow cannula 77112, 9”, 12Fr), both Systems A and B delivered 1.2–1.5 L/min flow against a ΔP circuit of 50-75mmHg at 5000 RPM (**Fig 3C and 3D**) and up to 1.4 L/min flow against a ΔP pump of 240mmHg at 5000 RPM (**Fig 4C and 4D**). In the 2400mL volume mock loop (largest cannulae pair: inflow cannula 68124, 15”, 24Fr; outflow cannula 77116, 9”, 16Fr), both Systems A and B delivered up to 2.2 L/min flow against a ΔP circuit of 50-75mmHg at 5000 RPM (**Fig 3E and 3F**) and up to 2.2 L/min flow against a ΔP pump of 230mmHg at 5000 RPM (**Fig 4E and 4F**).

Intrinsic pump parameters were recorded during all six static mock loop experiments and included motor current, voltage, and temperature. Mean temperature of the pump motor for both Systems A and B during testing of each of the three cannula pairs averaged 25.8°C at 500 RPM and increased to 60.5°C at 5000 RPM. Mean power use averaged 0.3 W at 500 RPM and increased to 40.4 W at 5000 RPM. Temperature and power use were consistent across RPMs regardless of the cannula pair tested.

### Dynamic mock loop model

**Fig 5:**
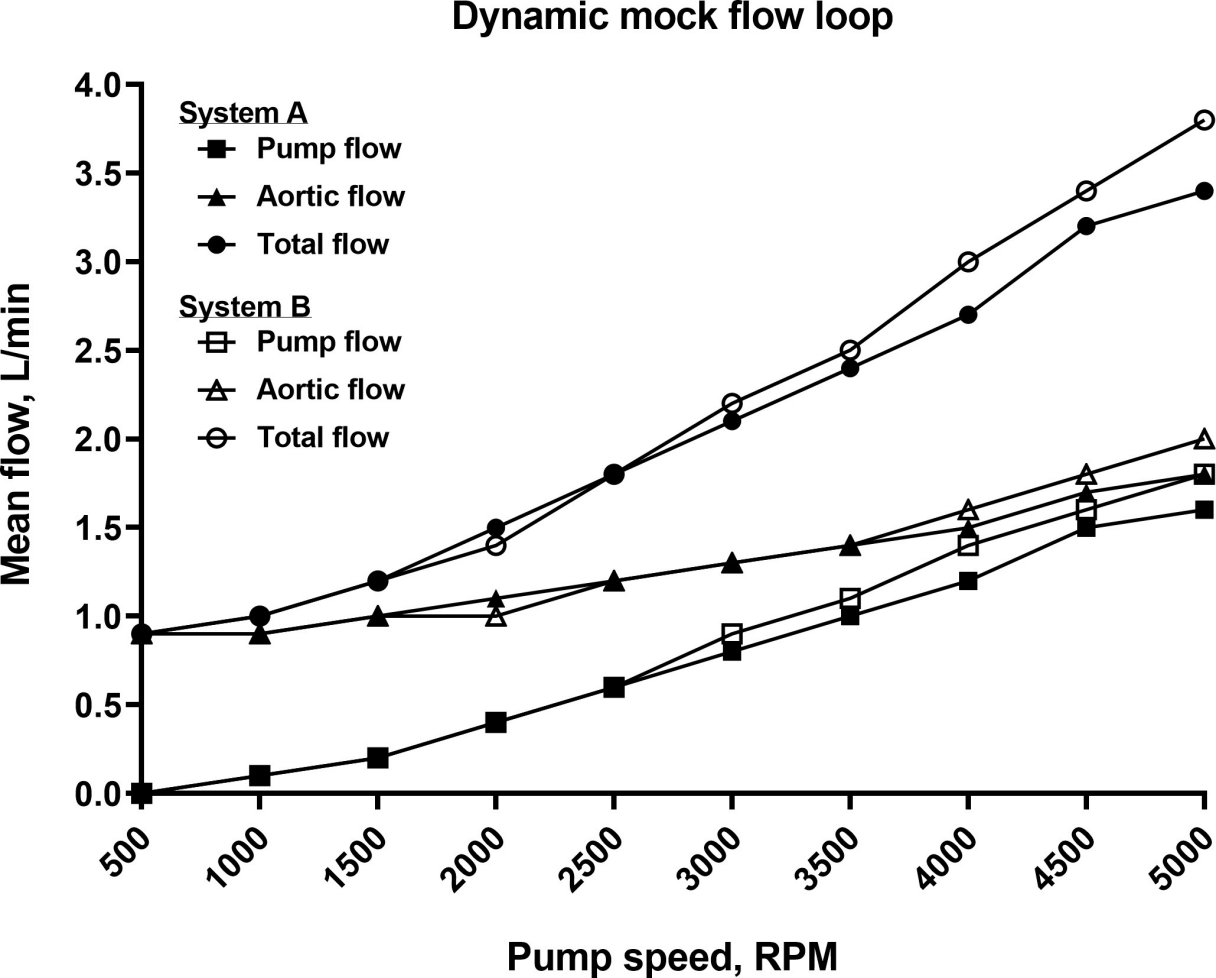
Dynamic mock flow loop data. Relationship between pump flow, mock ventricular flow, and total mean flow for the Inspired Therapeutics NeoMate System and inflow (24F) and outflow (16F) cannula in a dynamic mock flow loop model over a range of pump speeds for Systems A and B. These data show increasing mean flows and diminishing pulsatility with increasing pump speeds.

Experiments were performed using a pneumatic mock left-sided ventricle to quantify *in vitro* hemodynamic performance of Systems A and B. The cannula pair tested was inflow cannula 68124, 15”, 24Fr; outflow cannula 77116, 9”, 16Fr using a 3600mL volume dynamic mock loop. The results from the two dynamic mock loop experiments demonstrated increasing flow with increasing pump speed (**Fig 5**). The hemodynamic efficacy of Systems A and B were comparable (**Table 1**). Intrinsic pump parameters were recorded during both dynamic mock loop experiments, which included motor current, voltage, and temperature. Mean temperature of the pump motor for both Systems A and B averaged 27.3°C at 500 RPM and increased to 54.5°C at 5000 RPM. Mean power use for both Systems A and B averaged 0.1 W at 500 RPM and increased to 43.9 W at 5000 RPM.

**Table 1:** Dynamic mock flow loop hemodynamic data.

System A	Pump off		RPM									
	Clamped	Unclamped	500	1000	1500	2000	2500	3000	3500	4000	4500	5000
**Pump inflow, mmHg**	45	46	45	44	40	35	27	18	8	-5	-1	-26
**Pump outflow, mmHg**	45	48	49	54	59	68	80	95	112	131	154	165
**ΔP pump, mmHg**	0	2	4	10	19	33	53	77	104	136	155	191
**LV pressure max, mmHg**	124	124	123	122	122	121	120	119	118	118	117	117
**LV pressure min, mmHg**	5	5	6	6	6	6	6	7	7	7	6	6
**LV end-diastolic pressure, mmHg**	18	18	18	19	19	19	18	17	17	17	16	16
**LV pressure mean, mmHg**	50	51	50	50	50	49	49	48	48	47	47	46
**Aortic pressure systolic, mmHg**	114	114	113	112	112	112	111	110	110	110	112	117
**Aortic pressure diastolic, mmHg**	34	33	32	35	37	44	49	54	56	60	68	71
**Aortic pressure mean, mmHg**	49	49	48	50	51	53	56	60	64	69	74	77
**Venous pressure, mmHg**	18	18	18	18	18	17	17	17	17	17	17	17
**Pump flow, L/min**	0	0	0	0.1	0.2	0.4	0.6	0.8	1.0	1.2	1.5	1.6
**Aortic flow, L/min**	0.9	0.9	0.9	0.9	1.0	1.1	1.2	1.3	1.4	1.5	1.7	1.8
**Total flow, L/min**	0.9	0.9	0.9	1.0	1.2	1.5	1.8	2.1	2.4	2.7	3.2	3.4
**System B**	**Pump off**		**RPM**									
	**Clamped**	**Unclamped**	**500**	**1000**	**1500**	**2000**	**2500**	**3000**	**3500**	**4000**	**4500**	**5000**
**Pump inflow, mmHg**	41	49	45	42	40	35	25	14	2	-13	-30	-30
**Pump outflow, mmHg**	46	47	49	51	56	65	82	100	120	142	169	169
**ΔP pump, mmHg**	5	-2	4	9	16	30	57	86	118	155	199	199
**LV pressure max, mmHg**	116	118	118	118	117	117	115	115	114	114	114	113
**LV pressure min, mmHg**	6	7	7	7	7	7	7	6	6	6	5	5
**LV end-diastolic pressure, mmHg**	17	17	18	18	18	18	18	16	17	16	15	14
**LV pressure mean, mmHg**	49	49	49	49	48	48	47	47	46	45	45	45
**Aortic pressure systolic, mmHg**	108	110	110	110	109	109	108	108	107	107	105	106
**Aortic pressure diastolic, mmHg**	30	31	31	35	38	45	50	54	58	65	73	81
**Aortic pressure mean, mmHg**	48	48	48	48	49	52	56	60	65	70	77	77
**Venous pressure, mmHg**	15	15	15	15	15	15	15	15	15	15	14	14
**Pump flow, L/min**	0	0	0	0.1	0.2	0.4	0.6	0.9	1.1	1.4	1.6	1.8
**Aortic flow, L/min**	0.9	0.9	0.9	0.9	1.0	1.0	1.2	1.3	1.4	1.6	1.8	2.0
**Total flow, L/min**	0.9	0.9	0.9	1.0	1.2	1.4	1.8	2.2	2.5	3.0	3.4	3.8

LV, left ventricle; ΔP, change in pressure.

### Bench testing

The third prototype (System C) demonstrated the ability to continuously provide 0.25 L/min flow against an average pressure head of 10 mmHg for an average of 8 hours/day for more than 365 days without system failures (pump, impeller, controller). The prototype to date continues to run Monday–Friday each week.

### Acute ovine model

**Fig 6:**
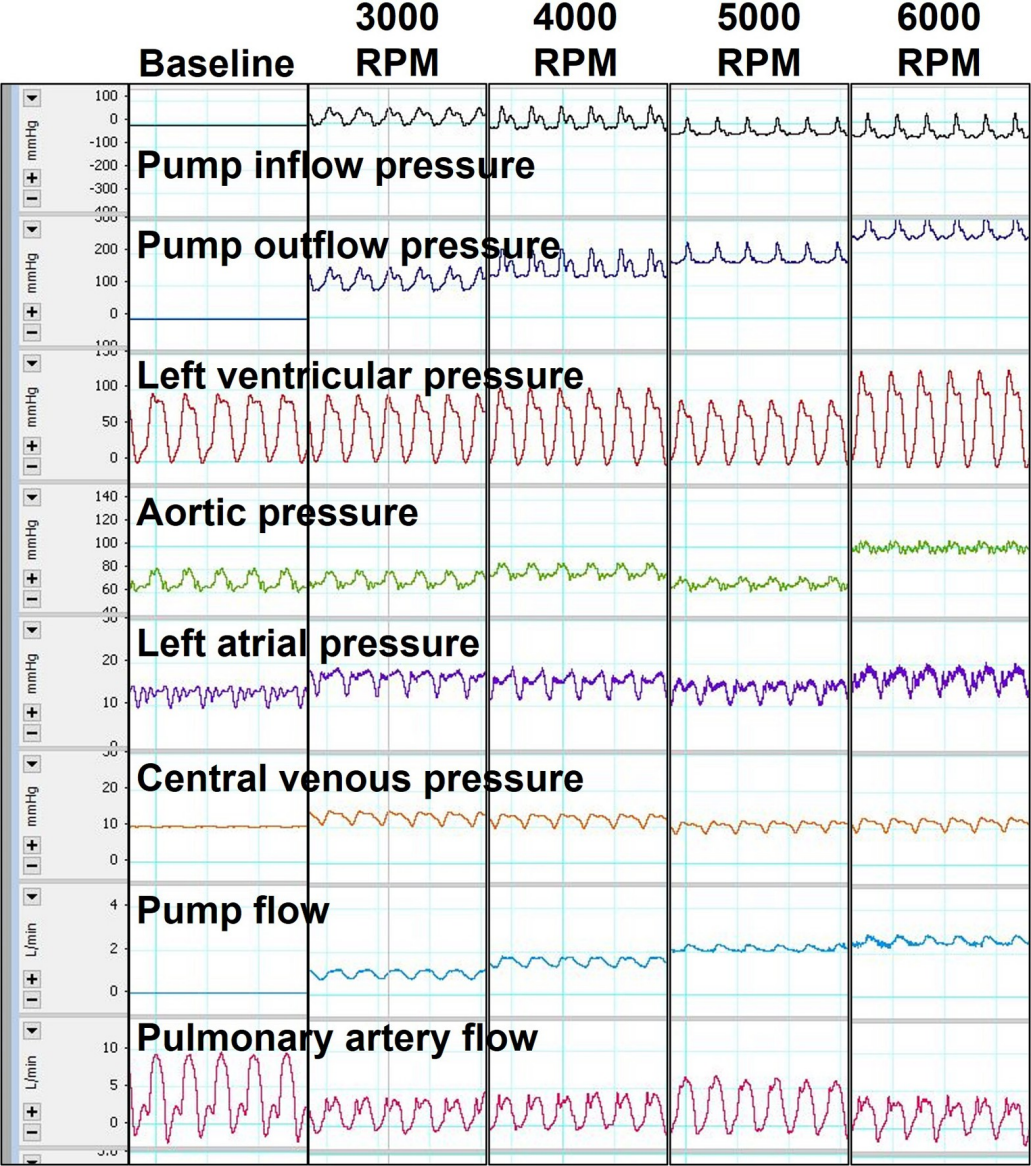
Hemodynamic waveforms. Representative hemodynamic waveforms recorded intraoperatively from one of the acute sheep (System A) at baseline (pre-pump implant, post-pump implant off and clamped/unclamped) and at increasing pump speeds.

**Fig 7:**
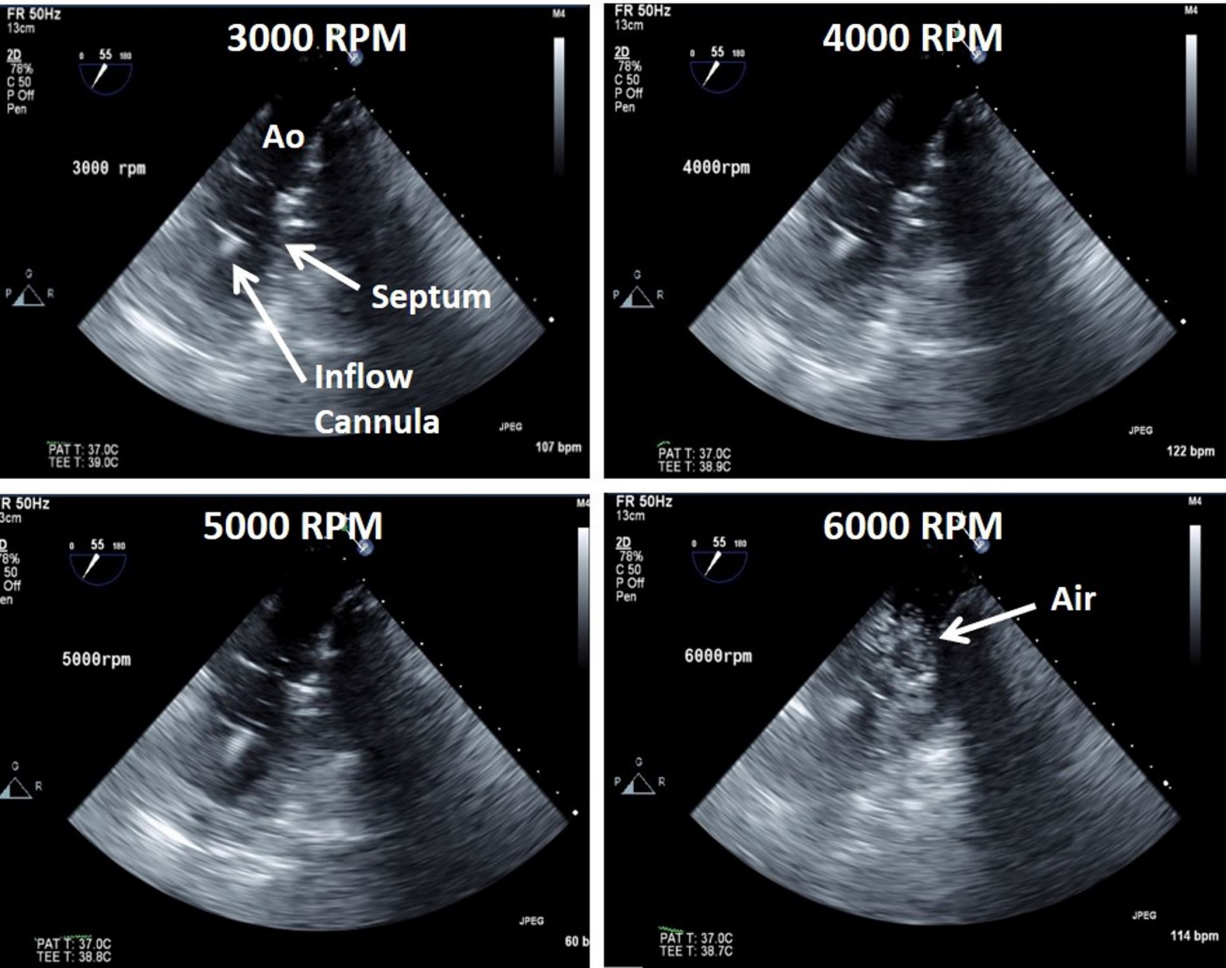
Echocardiography. Transesophageal echocardiographic (TEE) images obtained via a modified 4-chamber view from one of the acute sheep (System B) showing leftward intraventricular septal shift with increasing pump speeds. Note the presence of air emboli visible at 6000 RPM resulting from challenges with de-airing the pump.

Two acute (non-survival) sheep experiments were performed to assess pump performance of Systems A and B to demonstrate early prototype system feasibility *in vivo*. The experimental procedure, including instrumentation, surgery, pump implant, and execution of testing parameters were completed in both animals. Baseline bloodwork and hemodynamics (pre-LVAD implant) were consistent with normal healthy animals. The animals each had a mean aortic pressure of 80 mmHg and as measured via transesophageal echocardiography, the animals had a left ventricular ejection fraction (EF) of 60% and a mean cardiac output (CO) of 6.7 L/min (**Table 2)**. Pulmonary artery flow measured by clamp-on transit-time flow probe (**Table 2**) did not match the transesophageal echocardiography-based (TEE) CO calculations, which may be due to flow probe measurement error (air bubbles, poor fit, coupling mismatch). Initiation of pump support and acquisition of data at 3000, 4000, 5000, and 6000 RPM was completed (**Fig 6**). Pump flow (1.0–2.6 L/min) increased linearly with increasing pump speed (3000–6000 RPM). Increasing LVAD support also resulted in progressive decreases in aortic pulse pressure and left ventricular volume (**Table 2**). The interventricular septum, as documented via TEE using a modified 4 chamber view, appeared to bow more toward the left ventricle with increasing LVAD speeds, suggesting volume unloading (**Fig 7),** and no suction events were observed. ACT was >400s throughout the duration of testing in both animals. Challenges with de-airing the pump resulted in air emboli in the left ventricle and aorta that were documented via TEE (**Fig 7**). Periodic arrhythmias and a reduction in blood pressure were observed and as a result phenylephrine (100mcg/mL IV) was administered to both animals intraoperatively to support cardiovascular function. Plasma free hemoglobin measured 0 at baseline and remained 0 in both animals during LVAD support. This result was a little surprising given the early prototype stage of device development but may be due in part to the relatively short and acute duration of pump support during acute animal testing. As this is the very first feasibility testing of an early prototype device, hemolysis testing and hemocompatibility assessments were not performed at this time; only surgical feasibility, hemodynamic assessment, and intrinsic pump parameter assessment. Hemolysis testing and hemocompatibility testing are future objectives that will be completed prior to system design freeze to demonstrate ASTM standards achieved.

**Table 2:** Summary of hemodynamic data.

	Sheep 1, System A							Sheep 2, System B						
		LVAD implanted							LVAD implanted					
		Pump Off	Pump Off	RPM	RPM	RPM	RPM		Pump Off	Pump Off	RPM	RPM	RPM	RPM
	BL	Clamped	Unclamped	3000	4000	5000	6000	BL	Clamped	Unclamped	3000	4000	5000	6000
**Pump inflow pressure, mmHg**	x	60	41	27	7	-32	-41	x	42	46	21	-41	-97	-180
**Pump outflow pressure, mmHg**	x	70	49	112	151	182	261	x	45	57	116	122	150	142
**LV pressure max, mmHg**	95	89	73	94	102	86	127	92	98	88	96	78	99	94
**LV pressure min, mmHg**	-2	-4	5	-1.7	-5	-4	-8	4	4	5	7	5	3	2
**LV pressure mean, mmHg**	47	41	36	47	50	42	61	43	40	36	39	31	36	31
**LV end-diastolic pressure, mmHg**	20	6	14	11	10	12	13	11	10	10	11	11	9	10
**Aortic pressure systolic, mmHg**	81	71	65	79	85	74	105	79	81	75	80	68	81	76
**Aortic pressure diastolic, mmHg**	60	52	44	63	70	62	94	65	64	54	67	55	69	63
**Aortic pressure mean, mmHg**	69	61	51	71	77	68	99	71	70	61	72	59	73	67
**Aortic pulse pressure, mmHg**	21	19	21	16	15	13	11	14	18	21	13	12	13	13
**Left atrial pressure, mmHg**	13	14	16	16	16	14	17	13	12	12	13	13	12	12
**Central venous pressure, mmHg**	10	11	12	13	12	11	12	4	5	4	0	0	1	1
**Carotid artery pressure, mmHg**	62	54	46	66	72	61	92	62	61	52	67	51	61	58
**Heart rate, bpm**	95	107	103	103	104	103	105	129	93	89	89	87	85	85
**Pump flow, L/min**	x	0	-0.3	1.0	1.5	2.1	2.6	x	0	-0.4	0.8	1.2	1.3	x
**Pulmonary artery flow, L/min** *	3.8	2.1	1.1	1.7	2.0	2.7	1.7	2.4	2.5	2.6	3.0	2.1	2.9	2.7
**LV volume, mL**	x	x	x	x	x	x	x	20	x	x	17	14	15	12

*Note the pulmonary artery flow measured by flow probe did not match the transesophageal echocardiography-based (TEE) calculations which may be due to flow probe measurement error (air bubbles, poor fit, coupling mismatch).

Intrinsic pump parameters were recorded intraoperatively during both acute sheep studies and included motor current, voltage, and temperature. Temperature of the pump motor averaged 33.6°C in both animals at 3000 RPM, and temperature increased linearly with increasing RPM, measuring 62°C in both animals at 6000 RPM. Power use of the pump and controller averaged 9.8 W in both animals at 3000 RPM and increased to 48.1 W at 6000 RPM.

**Fig 8:**
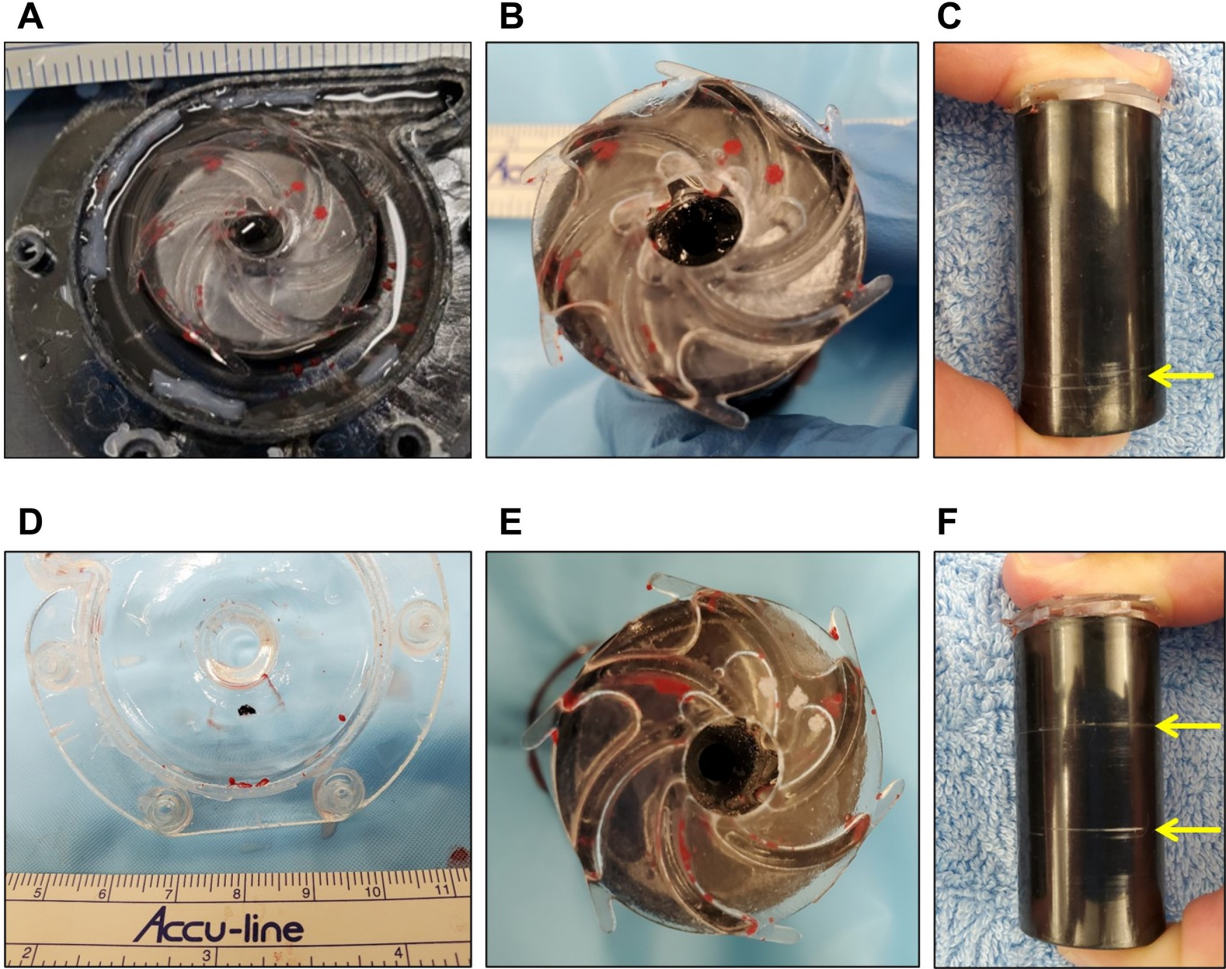
Pump explant images. Photographs of the pump impellers, housing, and magnets at necropsy following the acute ovine model experiments for System A (panels A-C) and System B (panels D-F). Panels A, B, D, and E show the presence of small thrombi on the pump housing and under the impellers of both systems. Panels C and F show the presence of wear (yellow arrows) on the magnets. Note that in panels A and B, the pair of red dots at the 1 and 2 o’clock positions are not thrombi but ink dots marking the identity of the specific impeller used.

Necropsies were performed on both animals following conclusion of the acute testing. The heart, great vessels, and end organs of both animals were grossly normal with no evidence of thrombus, bleeding, or other notable concerns. There were no gross signs of thrombus in the inflow or outflow cannulae of either animal. The pump and controller were carefully disassembled, rinsed gently with saline, and examined for the presence of thrombus, wear, or other notable features. As shown in **Fig 8**, small thrombi were observed under the impeller of System A, and wear was present on the lower portion of the magnet. In System B, small thrombi were observed on the pump housing and under the impeller, and wear was present in two places along the middle portion of the magnet.

The long-term vision of Inspired Therapeutics is to provide disposable, low-cost pumps for use with a single, multi-functional, universal controller to support multiple pediatric cardiopulmonary indications and that is capable of being used in any hospital setting. The small size and weight of the system is designed to facilitate mobility and transport of the patients. LVAD therapy has been shown to be effective in supporting the <10kg HF population to heart transplant [15]; however, MCS options available to these patients are limited. In response to this critical clinical need, Inspired Therapeutics is focusing on the underserved neonate and infant HF patient population for initial application of the NeoMate System.

In this study, we completed proof-of-concept and feasibility testing of the early prototype NeoMate System in static mock flow loops, dynamic mock flow loops, and acute animal models in order to generate useful information to facilitate design improvements for the next iteration of the system. Although the desired target flow rate of up to 3.5 L/min at 6500 RPM pump speed was not achieved, flow rates of up to 0.7 L/min, 1.4 L/min, and 2.2 L/min for the smallest to largest cannula pairs (static mock loop) at 5000 RPM, 1.7 L/min at 5000 RPM (dynamic mock loop), and 2.6 L/min at 6000 RPM (healthy acute animals) were accomplished. No gross device or controller failures, such as break, leak, and/or malfunction, occurred during experimental testing. Small thrombi were present on the pump housing and under the impellers of both systems at necropsy. While the underlying causes and mechanisms are presently unknown, we speculate they could be the result of the prototype 3D printed parts being imperfect (rough edges and/or textures, porous material), increased motor temperature contributing to thermal effects on the blood, and/or mechanical imbalance (as evidenced by wear on the magnets) which may have created areas of stasis.

### Limitations and challenges identified

Using the results from these early-stage prototype testing studies and during the development process itself, several NeoMate System features requiring design improvements were identified. First, the prototype pumps were 3D printed (SLA) and manually assembled, which may have resulted in imperfections in the pump geometry and flow paths. Similarly, imperfections in the impellers (sharp edges, material drips/build up, poor feature reproduction) may have contributed to reduced impeller efficiency and subsequent lower flow rates. SLA 3D printing techniques have several limitations including surface roughness of the material, increased material porosity compared with injection molded materials, and sub-par detail definition (radii of edges). For the next design iteration injection molded components and a more precise radii definition and assembly method will be used. Second, the rotating group assembly was not mechanically balanced and exhibited some vibration at the higher speeds, which may have led to issues in the motor drive control loops, such as inaccuracies in the commutation switch points, which resulted in lower effective torque constants and higher torque ripple. Third, the motor temperature and power increased with increasing pump speed, likely due to rotating group assembly imbalance causing mechanical vibrations, which not only draw additional power, but also adversely affect the operation of the control loops designed to rotate the rotor at a desired speed. Higher power consumption and winding temperatures are a function of motor design and efficiency (e.g. gap spacing, magnets used, coil construction, operating point) along with the hydraulic efficiency of the pump and impeller design. The gap spacing between the rotating magnet assembly and motor windings is large (0.090”-0.100”) in this early prototype due to the thicknesses of housings (rotor body, lower pump housing) and a large radial blood gap between the rotor and stator. Although high motor winding temperatures of up to 60°C were observed under some test conditions, mock flow loop fluid temperature was maintained at 37°C in all experiments and no increase in hemolysis was measured during acute animal experiments, indicating motor winding temperature may not have an acute effect on the pump fluid volume. Further CFD analysis and experiments are needed to explore the effects of motor temperature on the flow of blood through the pump housing. It is anticipated that future design iterations for motor gap spacing, hydraulic efficiency of the impeller, and use of a back-iron in the motor design will potentially reduce maximum winding temperatures by 70%.

During experimental testing, operational challenges requiring design improvements were also identified. First, it was difficult to completely and efficiently de-air the pump, tubing, and cannulae due to assembly and integration of the pump and MagLev system into the controller (ex. unable to see air trapped under the impeller and rotor body), the length of tubing used, and the use of tubing connectors. Second, mechanical vibrations and noise at higher pumps speeds (> 4500 RPM) were observed, which may be due to mechanical imbalance of the rotating assembly that caused instability or inefficiency with the MagLev system. Additionally, PID loop parameters Kp, Ki, and Kd for longitudinal position control were selected to provide positional stability at all pump operating points while minimizing VCA current and related power dissipation but may require fine-tuning. Third, small thrombi were present on the pump housing and under the impellers of both controllers in the acute animal experiments.

### Potential solutions

To address these issues, the following potential solution(s) are being carefully considered for the next design iteration. First, integration of the pump, motor, and controller into a single compact unit that will be small in size (51 mm diameter by 115 mm height, 27mm diameter impeller, 681g weight) to allow for pump movement and manipulation during de-airing, (2) reduction of rotor body height which will reduce the amount of radial blood gap area where air can be trapped inside the pump housing and to potentially improve de-airing, (3) re-design the rotating magnetic group to be dynamically and mechanically balanced with careful selection of magnetic components, automation of rotor assembly, and implementation of a method to harmonically balance the rotor assembly to eliminate vibration and contact with the pump housing walls, and (4) dynamic balancing of the rotor will require the addition of features to accommodate material removal during the dynamic balance operation. The next generation design will use blood as a hydro-dynamic bearing fluid, which will require further analysis of the balancing of magnetic radial forces with the counter forces supplied by the gap fluid. Additional design improvements may include: 1) reduction in pump size by implementing dual-lamina construction integrated into the pump housing; 2) shortening the circuit length by separating and placing the pump closer to the recipient. This has the benefit of facilitating the de-airing process, eliminating connectors (which may contribute to thrombus formation), minimizing blood heat loss through the tubing, and minimizing the blood contact foreign surface area; 3) improve motor, stator, and VCA winding geometries to improve efficiency and reduce power dissipation and radio frequency (RF) interference; 4) a secondary microcontroller for VCA will be implemented to control the longitudinal position of the rotor/impeller and the driver using a fully integrated H-Bridge driver.

## Conclusions

In conclusion, we developed and fabricated prototypes of the Inspired Therapeutics NeoMate System and performed feasibility testing in static mock flow loops, dynamic mock flow loops, and acute animals. The resultant hydrodynamic and hemodynamic data demonstrated the ability of the system to function over a range of rotary pump speeds (500–6000 RPM), to provide pump flow rates of up to 2.6 L/min, and to volume unload the left ventricle in acute animals. No gross device or controller failures, such as break, leak, and/or malfunction, occurred during experimental testing. Key engineering challenges observed and proposed solutions for the next design iteration were identified and will be used to facilitate design improvements for the next iteration of the NeoMate System.
